# Co-occurrence of bilateral nodular anterior scleritis and large-vessel arteritis in a patient with TINU syndrome

**DOI:** 10.3205/oc000123

**Published:** 2019-10-25

**Authors:** Kirsten Zonnevylle, Pieter-Paul Schauwvlieghe, Joachim Van Calster, Jan Lenaerts, Domien Peeters

**Affiliations:** 1 Ophthalmology, University Hospitals Leuven, Belgium; 2 Department of Rheumatology, University Hospitals Leuven, KU Leuven, Belgium; 3 Reuma-Instituut Hasselt, Belgium; 4 Nephrology, Sint Trudo Hospital, Sint-Truiden, Belgium

**Keywords:** interstitial nephritis, uveitis, tubulointerstitial nephritis and uveitis syndrome, scleritis, arteritis

## Abstract

We present a case of tubulointerstitial nephritis and uveitis (TINU) with nodular anterior scleritis and large-vessel arteritis. A 67-year-old patient was admitted to the hospital with high fever, thoracic pain, and weakness. Bilateral anterior uveitis was seen at that time. Laboratory examination showed acute renal failure. A renal biopsy was performed and showed pathognomonic signs of tubulointerstitial nephritis (TIN). Six months later, she developed ocular inflammation suggestive of nodular scleritis. One year after hospital admission, she presented with large-vessel arteritis. We describe a case of TINU with co-occurrence of scleritis and large-vessel arteritis.

## Introduction

We present a case of a 67-year-old patient with biopsy-proven interstitial nephritis and bilateral uveitis. The diagnosis of TINU syndrome was made. She developed a nodular scleritis six months later and presented one year after hospital admission with large-vessel arteritis. The co-occurrence of nodular scleritis in a patient developing tubulointerstitial nephritis and uveitis (TINU) has been described only once before [1]. There are no reports of TINU syndrome with large-vessel arteritis. This case illustrates the possibility that nodular scleritis and/or large-vessel arteritis may co-exist with TINU syndrome, albeit with a time lag between the different diseases.

## Case description

**Figure 1:**
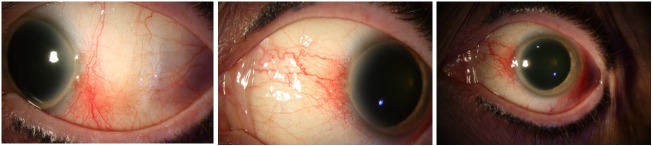
The anterior segment of both eyes revealed a nasally and temporally sectoral conjunctival injection with a nodule suggestive of nodular anterior scleritis.

**Figure 2:**
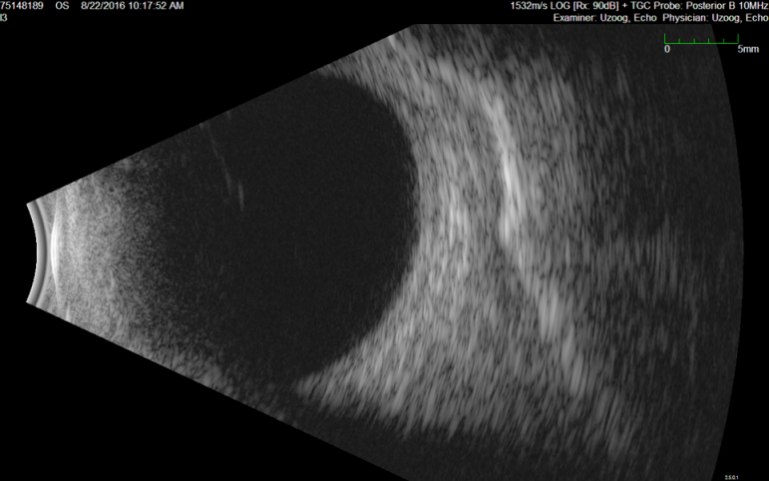
B-scan ultrasonography revealed no fluid in Tenon’s capsule, excluding posterior scleritis.

In 2016, the patient presented with general weakness, thoracic pain, and high fever. The patient was initially diagnosed with a bacterial infection and antibiotics (Amoxicillin) were started. However, the fever persisted, and she developed an acute renal insufficiency. A urinalysis indicated a creatinine level of 2.7 mg/dl and proteinuria (1.4 mg/mg creatinine). Further examinations were performed to determine the underlying systemic cause [2], [3]. Testing included a chest radiograph, classic serology for auto-immune diseases (antinuclear antibodies (ANA) and anti-neutrophil cytoplasmic antibodies (ANCA) testing), complete blood count, serum creatinine, and the erythrocyte sedimentation rate (ESR). No underlying systemic illness was found. A renal biopsy was performed, which showed an acute tubulointerstitial nephritis. Fortunately, renal function recovered spontaneously without systemic treatment. Ophthalmologic examination showed bilateral anterior uveitis and treatment with topical steroids was started. Six months later, she was referred to the ophthalmology department of our hospital because of recurrent ocular inflammation. Her best-corrected visual acuity (BCVA) was 10/10 in both eyes. The anterior segment of both eyes revealed a nasally and temporally sectoral conjunctival injection with a nodule suggestive of a nodular anterior scleritis (Figure 1 (Fig. 1)). The anterior chamber and fundoscopic exams of both eyes were unremarkable. B-scan ultrasonography revealed no fluid in Tenon’s capsule, excluding posterior scleritis (Figure 2 (Fig. 2)). The superficial vessels did not blanch with 10% phenylephrine, which excluded episcleritis. 

### Treatment 

A treatment for this scleritis was started with topical steroids q.i.d. Due to the possibility of recurrent ocular inflammation, immune-suppressive therapy was advised. However, there was a good response on topical treatment and the renal function normalized. Therefore, systemic steroids were not given. 

### Outcome and follow-up 

The patient underwent a follow-up evaluation three months after her first consultation at our department. She had been using topical steroids for a few weeks. The examination revealed an improvement of the nasally and temporally sectoral conjunctival injection and no intra-ocular inflammation. Furthermore, pain and discomfort had diminished. Therefore, no topical or systemic medication was further prescribed. One year after hospital admission, the patient presented with general extreme fatigue. A rheumatological examination showed signs of polymyalgia rheumatica, and subsequent serology testing showed intense elevated inflammatory parameters (ESR of 102 mm/h and C-reactive protein level of 76 mg/l). Based on these findings, a PET CT scan was performed, which showed well-defined vasculitis [4]: increased FDG (fluorodeoxyglucose) avidity was present in the aorta, brachiocephalic trunk, subclavian and carotid arteries, and iliac arteries up to the tibial arteries. Systemic steroids (methylprednisolone 64 mg daily) were initiated and a biopsy of the temporal artery was performed. Microscopic examination of this biopsy revealed intimal hyperplasia of the arterial wall, diffuse infiltration of mononuclear cells and fragmentation of the internal elastic lamina. No multinucleated giant cells were seen in this biopsy. The combination of systemic inflammation, PET results in combination with the clinical picture, and the temporal artery pathological findings were very suggestive of large-vessel vasculitis.

## Discussion

TINU syndrome was first described in 1975 by Dobrin and associates [5]. They described two patients with acute interstitial nephritis, uveitis and bone marrow granulomas. To date, approximately 250 cases of TINU syndrome have been reported worldwide. Renal and ocular symptoms are not always clinically evident at the same time. Therefore, the diagnosis of TINU syndrome is not always considered. The cause of TINU syndrome is not known. Possible etiologies of interstitial nephritis include drugs, infections, and auto-immune diseases [6], [7]. However, in 10% of all patients, interstitial nephritis is caused by TINU syndrome. Therefore, it is very important to consider this disorder in patients presenting with acute interstitial nephritis or idiopathic bilateral anterior uveitis.

Mandeville et al. reviewed 133 case reports of TINU syndrome and reported an overview of all clinical features [8]. The clinical presentation was highly variable and affected mainly women between the first and second decade of life. The characteristic ocular symptoms were eye pain, redness, blurred vision, and photophobia, hence characteristics of an anterior uveitis. Slit lamp examinations typically revealed anterior non-granulomatous uveitis, but intermediate and posterior uveitis were described as well. The common systemic symptoms were fever, fatigue, malaise, and weight loss.

Ocular symptoms preceded systemic symptoms in 21% of the cases, and 65% of patients developed ocular symptoms after the initial systemic symptoms. 

Only one case report described anterior nodular scleritis with TINU syndrome before [1]. Repeated episodes of scleritis preceded the development of TINU syndrome in this patient. Treatment with oral prednisone was started for a period of six months and was successful. Considering the case report of Daniel et al. and taking into account the results of this case described above, it is important to consider ocular manifestations other than uveitis when TINU syndrome is considered. 

The co-occurrence of large-vessel arteritis and TINU syndrome has not been reported yet although there was a time lag between the two diseases. A literature search showed one article describing four case reports of (epi)scleritis an giant-cell arteritis together without underlying TIN [9]. The time interval in these cases between (epi)scleritis and subsequent giant-cell arteritis was much shorter than in our patient. We report a case of a middle-aged woman presenting with both scleritis and co-existing large-vessel arteritis after an initial diagnosis of acute tubulointerstitial nephritis. 

## Conclusions

To our knowledge, this is the first report of a case describing a patient with TINU syndrome, nodular scleritis, and co-existing large-vessel arteritis. Review of the literature shows that nodular scleritis may be part of the inflammatory spectrum seen with TINU syndrome or giant-cell arteritis. However, we cannot conclude that large-vessel arteritis is part of the inflammatory spectrum seen with TINU syndrome. In this case report, it rather reflects a simultaneous occurrence of large-vessel arteritis and TINU syndrome.

## Notes

### Competing interests

The authors declare that they have no competing interests.
